# Testing autonomous motivation as a mechanism of anticipated regret intervention techniques for seasonal flu vaccination

**DOI:** 10.1093/abm/kaaf053

**Published:** 2025-09-02

**Authors:** Austin S Baldwin, Catherine Rochefort, Alexander J Rothman

**Affiliations:** Department of Psychology, Southern Methodist University, Dallas, Texas, United States; VA Eastern Colorado Healthcare System, Aurora, Colorado, United States; Department of Psychology, University of Minnesota, Minneapolis, Minnesota, United States

**Keywords:** anticipated regret, autonomous motivation, vaccination, flu vaccine, experimental medicine

## Abstract

**Background:**

Anticipated regret is associated with vaccination. However, gaps in understanding mechanisms of anticipated regret and how to intervene using anticipated regret limit its use as an intervention to promote vaccination.

**Purpose:**

Address gaps in anticipated regret interventions to promote seasonal flu vaccination. In a randomized intervention, we tested the novel hypothesis that autonomous motivation is a target mechanism of anticipated regret and the effects of 2 techniques to elicit anticipated regret: self-generated and mere measurement.

**Methods:**

College students (*N* = 263) were randomized to complete (1) an open-ended anticipated regret prompt (self-generated), (2) anticipated regret questions (mere measurement), or (3) no anticipated regret questions or prompts. Participants then completed measures of motivation and vaccination intentions. The following spring, participants reported their vaccination status. Analyses were guided by the experimental medicine approach.

**Results:**

Self-generated anticipated regret led to greater autonomous motivation for vaccination (*d *= 0.39), evidence of target mechanism engagement. Self-generated anticipated regret also had a significant indirect effect on intentions (estimate = 0.335, 95% CI = 0.117-0.55) and vaccination (estimate = 0.035, 95% CI = 0.008-0.08) through autonomous motivation, evidence of target mechanism validation. However, neither anticipated regret intervention technique had a direct effect on intentions or vaccination.

**Conclusions:**

Autonomous motivation is a viable target mechanism of anticipated regret interventions to promote seasonal flu vaccination, and self-generated anticipated regret is an effective technique to engage autonomous motivation. Findings provide ample evidence for testing autonomous motivation as a mechanism of anticipated regret interventions in other contexts.

## Introduction

Vaccines to protect against infectious diseases are one of the great medical and public health achievements of the 20th century, yet vaccination rates are variable across different vaccines.^1^ Variability in vaccination rates is due, in part, to vaccine hesitancy, defined as the indecision and uncertainty about vaccination.^2^ Vaccine hesitancy has been identified as one of the top 10 threats to global health^3^ and has become an especially salient issue as a result of the COVID-19 pandemic.^2^^,^^4^ Vaccination against seasonal influenza was identified in *Healthy People 2030* as a Leading Health Indicator across all age groups to reduce flu infections and flu-related deaths.^5^ Yet, coverage rates for seasonal flu vaccines in the United States (2020-2021: 50.2%; 2021-2022: 49.4%; 2022-2023: 46.9%; 2023-2024: 48.7%;^6^) remain well below the *Healthy People 2030* target of 70%.^5^ Effective interventions to promote seasonal flu vaccination are needed.

One promising intervention technique to increase seasonal flu vaccination is to prompt individuals to consider their anticipated regret for not getting a flu shot. Anticipated regret is a cognitive expectation that elicits negative emotion in the moment.^7^^,^^8^ It involves imagining taking action (action regret), or choosing not to take action (inaction regret), that results in an unwanted future outcome. In the context of seasonal flu vaccination, inaction anticipated regret typically takes the form of imagining not getting a flu shot and then later getting sick with the flu. Anticipated regret is identified as a behavior change technique^9^ and is a promising approach to increase seasonal flu vaccination for multiple reasons. Anticipated regret is reliably associated with health behavior intentions and health behaviors, including vaccine intentions and vaccinations.^7^^,^^8^^,^^10^ Associations are particularly robust for inaction anticipated regret. In addition, anticipated regret is a stronger predictor of vaccination than other risk appraisals (e.g., severity, likelihood) and worry.^7^^,^^11^ Based on this prior evidence, we expect prompting individuals to consider inaction anticipated regret results in higher vaccination intentions and vaccination. Moreover, anticipated regret is a promising intervention technique because it requires little time and could be implemented in a variety of settings. Thus, it is highly scalable.

### Gaps in understanding anticipated regret as an intervention technique

There are critical gaps in understanding the effects of anticipated regret that currently limit its use as an intervention technique to promote vaccination. The Experimental Medicine Framework^12–14^ calls attention to the need to consider mechanisms to advance understanding of interventions. The Operating Conditions Framework^15^ builds upon the experimental medicine approach in its focus on the synergy between mechanisms and moderators and the conditions under which these components operate. Guided by these two frameworks, evidence for two key components is needed to advance understanding of how and why interventions work and to enhance the utility of intervention techniques. One, target mechanisms of the intervention for which there is evidence of a prospective association with the behavioral outcome, and two, effective intervention techniques that change the target mechanisms. The first gap is that mechanisms underlying the effect of anticipated regret on vaccination are unclear.^1^ Prior work in this area seems to assume that anticipated regret techniques (interventions) work because they evoke anticipated regret (mechanism), but this assumption has not been directly tested. As a result, little is understood about potential mechanisms anticipated regret interventions might evoke. Increased message involvement has been shown to mediate the effects of an anticipated regret intervention on HPV vaccine intentions,^16^ but there are no published studies to date that have tested mechanisms of anticipated regret interventions on vaccination behavior. The second gap is that there is a limited evidence base to guide how to effectively use anticipated regret as an intervention technique to promote vaccination. Most published studies examining the effect of anticipated regret are correlational in which individuals’ responses to anticipated regret measures are correlated with the outcome.^1^^,^^7^^,^^8^ To date, no anticipated regret intervention studies on vaccination have been published, and only a limited set of studies testing its effect in other domains.^17–20^ The objective of this study is to address these gaps in understanding the effect of anticipated regret as an intervention technique to promote seasonal flu vaccination.

We propose a novel hypothesis that autonomous motivation is a target mechanism through which anticipated regret influences vaccination. Self-determination theory^21^^,^^22^ posits two general types of self-regulation motivations: autonomous and controlled. Autonomous motivation for behavior reflects volition and the perception that the behavior is personally important, whereas controlled motivation for behavior reflects feeling pressure or coercion to engage in the behavior.^22^ Substantial evidence across various health behavior domains indicates behavior is more likely when it is autonomously motivated.^23^^,^^24^

The rationale for the novel hypothesis is based on theoretical arguments that anticipated regret might elicit greater autonomous motivation and on evidence for the prospective association between autonomous motivation and vaccination. In terms of the theoretical rationale, regret is a counterfactual emotion (ie, “I wish I would have chosen differently”) and is postulated to consist of two components: one associated with the undesired outcome, the other with recognizing one’s own volition in the choice.^25^ Moreover, regret is not experienced if one was not a causal agent in the decision to act or not.^26–28^  *Anticipated* regret prompts counterfactual thinking about future alternatives that implies volition between taking action or not (eg, getting a flu shot) and the future consequences of those choices (eg, likelihood of getting the flu). Anticipated regret is thought to be a strong motivator of behavior due to its focus on the evaluation of one’s own decisions and volition.^1^ Personal responsibility and volition are also central to autonomously motivated behavior.^22^^,^^29^ The provision of choice among alternatives has been shown to increase autonomous motivation across behavioral contexts^22^ and has been identified as an intervention technique to target autonomous motivation.^30^ Therefore, when individuals consider their anticipated regret of failing to take action (inaction regret) that would prevent a negative outcome, the provision of choice implied in the counterfactual thinking about the future might increase the salience of their personal volition and responsibility in the likelihood of the undesired outcome and their autonomous motivation to preventively act. In terms of prior evidence for the association between autonomous motivation and vaccination, studies on seasonal flu,^31^ adolescent HPV,^32^ and COVID-19 vaccines^33^ have demonstrated a prospective association between autonomous motivation and vaccination up to one year later. Therefore, we expect prompting inaction anticipated regret leads to greater autonomous motivation for vaccination, which in turn, should lead to an increase in vaccination.

Another critical gap is that how to intervene effectively using anticipated regret to promote vaccination is not yet well established. As noted previously, most studies on anticipated regret are correlational.^1^^,^^7^ Among the few studies that have tested anticipated regret as an intervention technique to change health behavior, it has typically been manipulated by randomizing whether individuals answer questionnaire items to assess anticipated regret or not.^16–19^ This approach is referred to as the mere measurement effect.^17^ Prior attempts using the mere measurement of anticipated regret as an intervention technique have largely proved unsuccessful in demonstrating a direct effect on behavior. Although one pilot study demonstrated a direct effect on organ donor registrations,^20^ other studies in cancer screening did not demonstrate a direct effect^17^^,^^19^ but instead effects conditional on other factors (eg, intention strength). Therefore, the mere measurement approach to prompting anticipated regret may not produce a robust direct effect.

Self-generated anticipated regret is a novel intervention approach to elicit anticipated regret. Self-generating reasons for engaging in health behaviors, including vaccinations, have been shown to result in greater cognitive engagement^32^ and in more personally relevant reasons for the behavior^34^ than reading or listening to arguments. Thus, prompting individuals to generate their own reasons they would regret not getting vaccinated could be more effective than responding to questionnaire items. An additional objective of this study is to conduct exploratory tests comparing the effects of self-generated anticipated regret and the mere measurement of anticipated regret on autonomous motivation for vaccination, intentions, and vaccination.

### Current study

**Figure 1.**
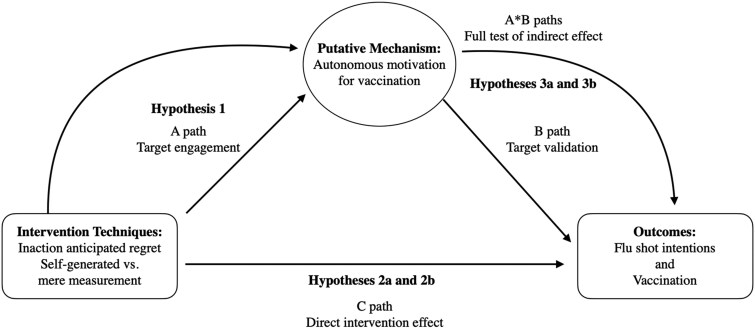
Hypothesized effects of the anticipated regret intervention techniques on target mechanism and outcomes.

We sought to address important gaps in understanding the effects of anticipated regret as an intervention technique to target seasonal flu vaccination. In Fall 2020, we randomized participants who had not yet received a flu shot to one of three conditions: self-generated anticipated regret, mere measurement of anticipated regret, or no anticipated regret questions or prompts. Following the anticipated regret intervention, participants completed measures of motivation and vaccination intentions, and then in Spring 2021, self-reported flu vaccination. Hypotheses based on prior evidence and theory led to multiple predictions. The hypotheses also map to tests of target engagement, target validation, the direct intervention effect, and the full test of the target mechanism pathway as outlined in the experimental medicine framework.^12^ First, we expect the anticipated regret intervention for the seasonal flu vaccine lead to greater autonomous motivation for vaccination (Hypothesis 1), a test of target engagement (Figure 1, A path). Second, we expect the anticipated regret intervention leads to higher flu shot intentions and vaccination (Hypotheses 2a and 2 b), tests of the direct intervention effect (Figure 1, C path). Third, we expect the anticipated regret intervention has an indirect effect on flu shot intentions (Hypothesis 3a) and vaccination (Hypothesis 3b) through autonomous motivation, tests of target validation and the full test of the target mechanism pathway (Figure 1, B path and A*B paths). Finally, we conducted exploratory tests to compare effects of self-generated anticipated regret and the mere measurement of anticipated regret on study outcomes.

## Methods

### Participants

Participants were college students (*N* = 263) at Southern Methodist University in Dallas, TX, who were recruited to participate in a study assessing beliefs about seasonal influenza and the flu shot. We excluded respondents who reported on a screening item that they had already received a flu shot for the current flu season (2020-2021). The flu vaccine was available to students at no cost if obtained through the university.

### Procedures

All study procedures, including informed consent, were administered online. The first part of the study was completed in Fall 2020. Participants in all three conditions were first provided general information about the seasonal flu and the flu shot (eg, common symptoms, single shot available in fall and winter months) and instructions specifically noting that upcoming questions were not asking about the coronavirus and COVID-19. This was to help ensure participants’ responses were in reference to the seasonal flu and flu shot and not COVID-19. After completing demographic information, participants were randomized to one of three conditions. The survey software (Qualtrics, Inc.) handled the randomization.

#### Self-generated anticipated regret condition

Participants (*n* = 89) responded to an open-ended prompt about inaction anticipated regret. The prompt read, “Imagine you get sick later in the year with the flu, but a flu shot would have prevented it. Why might you regret or feel sorry about that decision in the future?” Participants then provided their response in a text box.

#### Mere measurement of anticipated regret condition

Participants (*n* = 88) answered 6 anticipated regret questions. Two items were similar to inaction anticipated regret questions used in previous studies^11^^,^^35^ to which participants indicated their agreement with each statement (eg, “I would regret it I did not get a flu shot and later got sick with the flu”). Responses to these items (general negative consequences) were provided on 5-point scale ranging from *strongly disagree* to *strongly agree*. The two items were strongly correlated (*r* = 0.82). The other four items were developed following recommendations to create multi-item scales of anticipated regret by identifying multiple negative consequences of inaction.^7^ The instructions and stem to these four items (specific negative consequences items) read, “Imagine you get sick later in the year with the flu, but a flu shot would have prevented it. How much would you regret that you did not get a flu shot if you…” followed by four negative consequences (ie, have to spend several days in bed with unpleasant symptoms, end up spreading the flu to others, miss several of your classes, have to skip social activities). Responses were provided on a 4-point scale ranging from *Not at all* to *A lot*. The four items had strong inter-item reliability (*α* = .83).

#### Control condition

Participants (*n* = 86) did not receive anticipated regret prompts or questions after seeing the general information and instructions to all participants.

After the intervention tasks, participants first completed measures of motivation for vaccination, then intentions to get a flu shot, and finally measures of other beliefs about the flu and vaccination. Only measures relevant to the present analyses are described below. The second part of the study was completed between January and April of 2021, in which participants who had completed the first part were asked to report their flu vaccination status from the prior fall (see Figure S1 for study assessment timeline). Most participants (84.0%) completed the follow-up questionnaire, and response rates were above 80% in all study conditions (82.0%, self-generated anticipated regret; 81.8%, mere measurement of anticipated regret; 88.4%, control).

### Measures

#### Responses to the self-generated anticipated regret prompt

Participant responses were coded by two independent raters for whether the response was relevant to the question prompt (yes/no), expression of regret (yes/no/mixed), and the topic of the reason given. Inter-rater agreement was high, with agreement on 92.4% of the codes (concordance rate: *M* = 0.92, SD = 0.16, Median = 1.00, Mode = 1.00), and discrepancies were resolved through discussion. Only two participants (2.2%) did not provide a relevant response to the prompt. Among participants who provided a relevant response, most expressed regret (87.4%) or a mix of regret and no regret (6.9%) in their responses. Five participants (5.7%) indicated no regret.

#### Motivation for vaccination

We used a version of the Treatment Self-Regulation Questionnaire (TSRQ) previously validated for HPV vaccination^36^ and adapted the wording for seasonal flu vaccination to assess autonomous, introjected, and external motivation. The stem was “The reason you would get a flu shot is because…” and four items assessed autonomous motivation (eg, “you want to take responsibility for your health”), two items assessed introjected motivation (eg, “you would feel guilty or ashamed if you did not”), and two items assessed external motivation (eg, “you feel pressure from others to do so”). Responses were given on a 1 (*strongly disagree*) to 5 (*strongly agree*) scale. Inter-item reliability was good for all three subscales (autonomous: *α* = .91; introjected: *α* = .76; external: *α* = .75).

#### Flu shot intentions

We used two items to assess participants’ intentions to get a flu shot (“I intend to get the flu shot this year” and “It is likely that I will get a flu shot this year”). The two items were strongly correlated (*r* = 0.91).

#### Vaccination status

In the follow-up questionnaire, participants reported whether they received a flu shot over the past several months. Responses were given as “Yes”, “No”, and “I don’t remember”. Responses of “I don’t remember” (*n* = 3) were recoded as “No”. Self-reported flu vaccine status has been shown to be a valid form of assessment.^37^^,^^38^

#### Prior year flu shot

In the initial questionnaire, participants reported whether they had received a flu shot the prior year (2019-2020). Responses were given as “Yes”, “No”, and “I don’t remember”. Responses of “I don’t remember” (*n* = 14) were recoded as “No”.

### Analysis plan

We examined demographic differences across groups with chi-square tests and ANOVAs. To test the first two sets of hypotheses about the effect of anticipated regret on autonomous motivation, flu shot intentions, and vaccination, we used linear (continuous outcomes) and logistic (vaccination) regression models with dummy-coded study condition variables. To test the third set of hypotheses about the indirect effect of anticipated regret through autonomous motivation, we used causal mediation models based on the potential outcomes framework for continuous and dichotomous outcomes, respectively.^39–42^ Causal mediation methods address the assumptions needed to make causal claims based on counterfactual conditions. We used the *mediation* package in R that allowed for multiple intervention groups and sensitivity analyses. We first tested the intervention-by-mediator interaction terms to test whether the effects of autonomous motivation on intentions and vaccination were moderated by intervention condition. If none of the interaction terms were statistically significant, we planned to estimate the models without the interaction terms.^41^ We ran sensitivity analyses^43^ to examine robustness of the indirect effects to violations of assumptions of no confounding of the mediator-to-outcome effect. All models included the prior year flu shot to control for its effect on the tests of study hypotheses.

The pre-registered hypotheses and analysis plan can be found at this link. The power analysis indicated a sample size of 318 participants to detect small-to-moderate effects (*d *= 0.35) on the outcomes (selected as a sufficiently meaningful effect size) with 80% power and an alpha level = 0.05. The final sample size deviated from the preregistered sample size. We enrolled throughout the fall but were unable to enroll more than 263 participants (ie, 55 short of target enrollment). Enrollment coincided with CDC-recommended timing for the flu shot (ie, fall months) and when the university flu shot campaign (eg, emails, flu shot clinics) occurred. In addition, the switch to different academic semesters created logistical challenges in continuing to enroll new participants while simultaneously conducting the follow-up with already enrolled participants. For these reasons, we opted to discontinue study enrollment in December 2020.

A small number of participants had average completion times for the motivation (*n* = 4) and intentions scales (*n* = 1) that were so short (ie, <1 second per item), it would have been highly unlikely for them to have read each item sufficiently. We re-ran the models with motivation and intentions as the outcomes after removing this small number of participants. There were no changes in the significance of the pattern of effects with these participants removed; therefore, we report the results of the models that include all participants (see Table S1 for results of models excluding these participants).

## Results

### Sample characteristics

**Figure 2.**
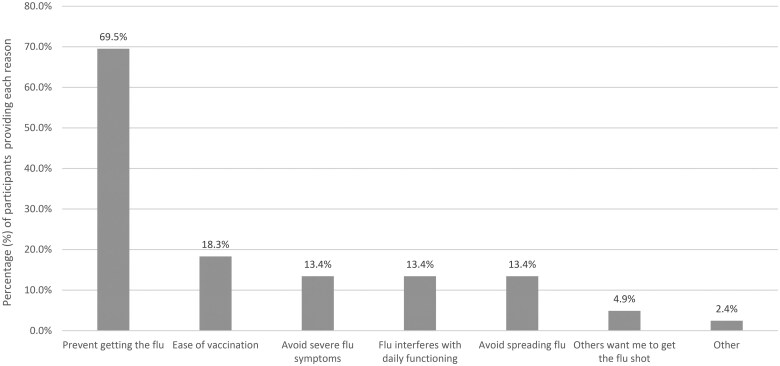
Specific reasons participants provided in response to anticipated regret prompt in self-generated anticipated regret condition.

Table 1 includes demographics and prior year vaccination rate for the entire sample and across the three study conditions. Information regarding sample socioeconomic status (SES) was not collected. There were no significant differences in the distribution of the sample characteristics or in the prior year vaccination rate across conditions. Figure 2 includes the specific reasons provided to the anticipated regret prompt from participants in the self-­generated anticipated regret condition. The percentages are based on the participants who reported regret in their response (82/89 participants) and do not sum to 100% across reasons because some participants provided multiple reasons.

**Table 1.** Sample characteristics for entire sample and by condition.

	Condition			
	Entire sample	Self-generated AR	Mere measurement of AR	Control
Sample characteristics	*N* = 263 (%)	*n *= 89 (%)	*n *= 88 (%)	*n *= 86 (%)
Gender				
Female	203 (77.2)	72 (80.9)	68 (77.3)	63 (73.3)
Male	60 (22.8)	17 (19.1)	20 (22.7)	23 (26.7)
Race/ethnicity				
White	191 (72.6)	67 (75.3)	61 (69.3)	63 (73.2)
Hispanic or Latinx	31 (11.8)	9 (10.1)	9 (10.2)	13 (15.1)
Asian or Asian American	21 (8.0)	7 (7.9)	10 (11.4)	4 (4.7)
Black or African American	13 (4.9)	4 (4.5)	6 (6.8)	3 (3.5)
Other	7 (2.7)	2 (2.2)	2 (2.3)	3 (3.5)
Age *M* (SD), range	19.6 (1.6); 18-33	19.6 (1.7), 18-33	19.6 (1.2), 18-24	19.8 (1.7), 18-29
Prior year vaccination rate^a^	0.50 (0.50)	0.45 (0.50)	0.59 (0.50)	0.48 (0.50)

There were no significant differences in the distribution of the sample characteristics across conditions.

Abbreviation:AR, anticipated regret.

a Prior year vaccination rate is the % who reported receiving the seasonal flu shot in the prior year.

### Reported experience of anticipated regret in intervention conditions

There is not a direct comparison of the effect of the anticipated regret manipulations across conditions. Instead, we report data on the experience of anticipated regret in the two intervention conditions. In the mere measurement condition, mean levels of anticipated regret on the two traditional anticipated regret items were 3.51 (SD = 1.32) and 3.48 (SD = 1.36), respectively (assessed on 5-point scales). These mean levels are higher relative to their scaling than anticipated regret reported in other studies, in which inaction anticipated regret for the flu shot was a significant predictor of intentions and vaccination.^11^^,^^44^ Correlations and descriptive statistics for all anticipated regret items in the mere measurement condition are included in Table S2. In the self-generated condition, as previously noted, 92.1% of participants reported some regret in their response to the open-ended prompt.

### Effects of anticipated regret on autonomous motivation

Results partially supported Hypothesis 1 that anticipated regret leads to greater autonomous motivation. Participants in the self-generated anticipated regret condition reported higher levels of autonomous motivation than those in the control condition (*β* = .188, *t *= 2.918, *P* = .004, *d *= 0.39 [95% CI, 0.09-0.69]). However, autonomous motivation levels did not differ between the mere measurement of anticipated regret condition and control (*β* = .051, *t *= 0.782, *P* = .435). In the exploratory analyses, autonomous motivation levels were significantly higher in the self-generated anticipated regret condition compared to the mere measurement condition (*β* = .138, *t *= 2.122, *P* = .035, *d *= 0.17 [95% CI: –0.12 to 0.47]). Means and standard deviations for autonomous motivation across the conditions are included in Table 2, along with descriptive information for other study outcomes.

**Table 2.** Means and standard deviations of study outcomes by condition.

	Condition		
	Self-generated AR	Mere measurement of AR	Control
Study outcomes	*M* (SD)	*M* (SD)	*M* (SD)
Autonomous motivation	3.88 (0.81)	3.72 (1.02)	3.53 (0.99)
Introjected motivation	2.74 (1.03)	2.72 (1.04)	2.54 (0.95)
External motivation	2.92 (1.06)	2.72 (1.01)	2.74 (1.15)
Vaccination intentions	3.63 (1.12)	3.48 (1.28)	3.49 (1.27)
Vaccination rate^a^	0.36 (0.48)	0.38 (0.49)	0.32 (0.47)

a Vaccination rate is the % who reported receiving the seasonal flu vaccine.

Abbreviation:AR, anticipated regret.

Although not included in the pre-registered hypotheses, we also tested the effects of anticipated regret on introjected and external motivation (two forms of controlled motivation) for comparison purposes. Neither anticipated regret condition led to differences in introjected (*β*s < .09, *P*s > .17) or external (*β*s < .08, *P*s > .24) motivation levels compared to control. There was also no difference between the two anticipated regret conditions (*β*s < .11, *P*s > .11). Correlations between measures of anticipated regret and motivation types in the mere measurement condition are included in Table S3.

### Effects of anticipated regret on flu shot intentions and vaccination

Results did not support Hypotheses 2a and 2b that anticipated regret would lead to greater intentions to get a flu shot and a greater likelihood of vaccination. Flu shot intentions were not significantly higher in either anticipated regret condition compared to control (*B*s < .07, *P*s < .28), and vaccination rates were not significantly higher in the self-generated anticipated regret (OR = 1.41; 95% CI, 0.68-2.94) or the mere measurement conditions (OR = 1.12; 95% CI, 0.54-2.31) compared to control. In the exploratory analyses, flu shot intentions were significantly higher in the self-generated anticipated regret condition compared to the mere measurement condition (*β* = .124, *t *= 1.995, *P* = .052, *d *= 0.12 [95% CI: –0.17 to 0.42]) but there were no significant differences between the two anticipated regret conditions in vaccination rates (OR = 1.26; 95% CI, 0.43-1.85).

### Indirect effects through autonomous motivation

We used causal mediation models^39–41^ to test whether anticipated regret had an indirect effect on flu shot intentions and vaccination through autonomous motivation (Hypotheses 3a and 3b). There were no significant intervention-by-mediator interaction terms on intentions or vaccination (*P*s > .33). Therefore, we tested the models without interaction terms via 10,000 bootstrapped samples. As seen in Table 3, the causal mediation effects were statistically significant for self-generated anticipated regret on intentions and vaccination compared to the control condition. The mere measurement anticipated regret condition did not have a significant causal mediation effect on either outcome. These findings indicate self-generated anticipated regret led to stronger flu shot intentions and a higher vaccination likelihood through increases in autonomous motivation compared to changes in autonomous motivation that would have occurred absent an anticipated regret prompt (ie, control). Results of supplementary analyses testing the association of autonomous motivation on vaccination adjusting for flu shot intentions, the initial outcome assessed, are in Table S4.

**Table 3.** Results of causal mediation tests of the indirect effects of anticipated regret through autonomous motivation.

	Dependent variable: flu shot intentions			Dependent variable: vaccination		
Indirect effect	Estimate	95% CI	*P*	Estimate	95% CI	*P*
ACME: self-generated AR versus control	**0.335**	**0.117 to 0.55**	**.003**	**0.035**	**0.008 to 0.08**	**.003**
ACME: mere measurement of AR versus control	0.091	−0.153 to 0.34	.46	0.013	−0.010 to 0.05	.28

Bold values indicate significant effects. Prior year vaccination was included as a covariate in these models.

Abbreviations: ACME, average causal mediated effect; AR, anticipated regret; CI, confidence interval.

We ran sensitivity analyses to examine the robustness of the indirect effects. Specifically, the analyses examine the expected mediated effect at different values of a hypothetical correlation (ρ) between an unmeasured confounder and both the mediator and the outcome. For flu shot intentions, the average causal mediated effect (ACME) would be rendered zero at ρ ≥ 0.71. This indicates a robust causal mediated effect as a very strong unmeasured confounder would be required to render the effect nonsignificant. For vaccination, the ACME would be rendered zero at ρ ≥ 0.21. Thus, the causal assumptions of this mediated effect would be violated in the case of an unmeasured confounder that has an even modest association with autonomous motivation or vaccination.

## Discussion

We tested the novel hypothesis of autonomous motivation as a mechanism of anticipated regret and compared the effects of two techniques to intervene using anticipated regret for flu vaccination (self-generated and mere measurement). Guided by the Experimental Medicine and Operating Conditions frameworks,^12–15^ tests of autonomous motivation as a mechanism of anticipated regret were partially supported. Self-generated anticipated regret led to greater autonomous motivation for vaccination, evidence of target engagement (Hypothesis 1). In addition, self-generated anticipated regret had a significant indirect effect on flu shot intentions and vaccination through autonomous motivation, evidence of target validation and the full test (Hypotheses 3a and 3b). However, neither anticipated regret intervention technique had a direct effect on flu shot intentions or vaccination (Hypotheses 2a and 2b). Also, the mere measurement of anticipated regret had no significant effects on the outcomes.

The findings are the first evidence to support the hypothesis that autonomous motivation is a mechanism of anticipated regret. To date, mechanisms evoked by anticipated regret have largely been untested.^1^ The findings suggest that when individuals actively consider their anticipated regret for inaction (eg, not getting a flu shot), it leads to greater autonomous motivation. In turn, greater autonomous motivation that results from considering anticipated regret leads to a higher likelihood of taking preventive action (eg, getting a flu shot).

Our findings also indicated that self-generated anticipated regret led to changes in autonomous motivation but not changes in introjected motivation or external motivation. It might be expected that anticipated regret would also elicit changes in introjected motivation, a type of motivation based in feelings of shame or guilt for not engaging in the behavior.^21^^,^^29^ However, prior evidence demonstrates regret is distinct from shame and guilt.^26^ Moreover, regret is based in decisions and behaviors involving personal volition and responsibility,^1^^,^^26^ whereas shame and guilt are based in decisions and behaviors influenced by the perceived expectations of others.^21^^,^^29^ Effects on introjected and external motivations for vaccination would be expected if an intervention technique made the expectations or influence of others’ (eg, healthcare providers, family members) salient to the decision to vaccinate.

In terms of the experimental medicine framework, the findings indicate that self-generated anticipated regret can be an effective intervention technique to engage autonomous motivation for vaccination. Several intervention techniques have been identified to target autonomous motivation.^30^ Anticipated regret is most closely related to the *Provide choice* technique (MBCT 6) in the taxonomy established by Teixeira and colleagues.^30^ Providing choice among alternatives has been shown to increase autonomous motivation across behavioral contexts.^22^ Although considering one’s anticipated regret does not explicitly provide choices between alternative options, regret is a counterfactual emotion (ie, “I wish I would have chosen differently”)^26^ and anticipated regret prompts counterfactual thinking about the future that implies choice between taking action or not and the future consequences of that choice. It is this provision of choice in influencing a future outcome that likely elicits autonomous motivation. Although the self-generated anticipated regret technique was brief and completed online in a single session, the effect on autonomous motivation (*d* = 0.39) was slightly larger than the average effect of different intervention techniques on autonomous motivation observed in meta-analyses (range: *d* = 0.23 to 0.30).^24^^,^^45^ In the context of vaccination, prompting individuals to actively consider their anticipated regret could be a more efficient approach to target autonomous motivation than other techniques. Using a brief intervention technique is also consistent with meta-analytic findings indicating that intervention intensity (eg, number of sessions, contact time) is not associated with intervention effect size targeting autonomous motivation.^45^

We observed a significant indirect effect of self-generated anticipated regret on flu shot intentions and vaccination, but contrary to our hypotheses, neither anticipated regret technique had a direct effect on intentions or vaccination. One possible explanation for this pattern is that the self-generated anticipated regret intervention, which was strong enough to affect autonomous motivation, was not strong enough to produce a direct effect on intentions or vaccination. In a meta-analysis of interventions with autonomous motivation as a target mechanism, the average intervention effect size for changes in autonomous motivation that resulted in significant behavior changes was substantially larger (*d* = 0.64)^46^ than the size of the effect in this study. Perhaps a stronger anticipated regret intervention (eg, additional open-ended anticipated regret question prompts), to produce even stronger effects on autonomous motivation, is needed to produce direct effects on vaccination. The lack of a direct effect is also consistent with several prior studies that have used anticipated regret as an intervention technique and have shown no direct effect of anticipated regret on behavior.^17^^,^^19^^,^^47^ In some cases,^17^^,^^19^^,^^48^ the effect of anticipated regret on behavior was conditional on moderating factors (eg, intention strength). It is possible the direct effect of self-generated anticipated regret is conditional on factors not measured in this study (eg, conscientiousness, neuroticism). In addition, behavioral intervention effects are stronger when the opportunity to engage in the behavior is temporally proximal to the intervention delivery.^49^ Testing this approach with a vaccination opportunity more temporally adjacent to the anticipated regret prompt may result in a stronger and direct effect. Future research should address these possibilities. Although there was not a direct effect of anticipated regret on vaccination, the indirect pathway through which anticipated regret influenced vaccination is a novel contribution. The findings also add to the growing evidence that autonomous motivation is a prospective predictor of vaccination,^31–33^ and thus an important target mechanism for vaccination.

The effects of anticipated regret on autonomous motivation and the indirect effects on intentions and vaccination were observed for the novel self-generated technique but not the mere measurement approach. There are multiple possible explanations for these differences. First, the self-generated technique may be a stronger manipulation of anticipated regret than mere measurement. Answering an open-ended prompt may elicit greater cognitive engagement in thinking about anticipated regret than answering questionnaire items. This is consistent with findings that self-generating reasons for vaccination resulted in greater cognitive engagement with the relevant content than listening to reasons for vaccination.^32^ A second related explanation is the self-generated technique may be an intervention strategy that changes autonomous motivation, whereas mere measurement may be a strategy that activates existing autonomous motivation but does not change it.^50^ We did not have measures of reaction time or baseline measures of autonomous motivation that would allow for tests to tease apart activation and change explanations. Future studies could include these features to directly address this possibility. Third, self-generated anticipated regret might produce reasons for anticipated regret that are more likely to be self-relevant and personally important than the reasons captured in questionnaire items.^34^ Reasons for vaccination that are self-relevant should be more likely to elicit autonomous motivation.^24^ The specific reasons participants generated for anticipated regret (Figure 2) largely overlapped in content with the reasons included in the questionnaire items, but also included a wider range of reasons including the *ease of vaccination* reason (cited by 18.3% of self-generated participants), *others want me to get the flu shot* reason (cited by 4.9%), and *other* reasons (cited by 2.4%), supporting this possible explanation. This explanation also suggests the possibility that autonomous motivation may be a mechanism specific to self-generated anticipated regret and not mere measurement. Future research is needed to clarify these possible explanations.

There are a few limitations of the current study. First, the sample size was smaller than what was pre-registered. The reason for this deviation from the pre-registration is that we stopped participant enrollment at the end of the calendar year (2020) to align enrollment with CDC-recommended timing for the flu shot and to accommodate the schedule for the follow-up assessments that began after the new year (January 2021). Although the resulting statistical power to detect the effect size we observed on autonomous motivation (*d* = 0.39) was slightly below the conventional threshold (estimated power = 0.75 rather than 0.80), the significant effects were consistent with the theoretically derived and preregistered hypotheses, which bolsters confidence in the pattern of findings. However, the smaller sample size calls for some caution in drawing firm conclusions about the true magnitude of the effects, including the nonsignificant effects (ie, direct intervention effects, all mere measurement effects). Second, the study was conducted with young adult college students in the context of decisions about the seasonal flu vaccine. The effects we observed may be different in other populations (eg, age groups for whom flu infection is more severe) or in decision-making about other vaccines. However, we would expect the effects of anticipated regret and autonomous motivation should generalize across different populations and vaccine contexts. Third, flu vaccination was self-reported rather than objectively assessed. The fact that self-reported flu vaccine status has been shown to be a valid form of assessment when compared to vaccine records^37^^,^^38^ tempers some concerns about this limitation. Fourth, the study was conducted during the initial months of the COVID-19 pandemic (but before COVID-19 vaccines were available). Although nationally in the United States, seasonal flu vaccine rates were similar in 2020-2021 to the preceding years,^6^ participants in our sample reported lower vaccination rates (34.8%) in 2020-2021 compared to the rate of flu shots the prior year (50%). Given the circumstances of when the study was conducted (eg, mix of students attending on-campus or remotely, prevention techniques of distancing and masking), it is possible that concerns about the seasonal flu were less salient in our sample that year and this could have influenced the effects we observed. Future research should investigate these effects in other samples and vaccine contexts. Fifth, we did not examine other potential mechanisms of anticipated regret interventions. For example, anticipated regret has been examined as an additional factor in the Theory of Planned Behavior (TPB).^8^ It is possible that the personal control and responsibility evoked by anticipated regret might have a similar effect through perceived behavioral control, a TPB construct, as the effect we observed through autonomous motivation.

## Conclusion

Autonomous motivation is a viable target mechanism of anticipated regret interventions to promote seasonal flu vaccination. In addition, self-generated anticipated regret is an effective intervention technique to successfully engage autonomous motivation for vaccination. Although there was no direct effect of self-generated anticipated regret on vaccination, testing this approach more temporally adjacent to a vaccination opportunity is needed to more clearly understand the strength of its direct effect. The effects indicating target engagement and validation of autonomous motivation as a mechanism of anticipated regret interventions provide ample evidence for testing its effects in other vaccination and health behavior contexts to establish their generalizability.

## Supplementary Material

kaaf053_Supplementary_Data

## Open science foundation (OSF) preregistration

The study was preregistered at the Open Science Framework (osf.io/p5u9j).

## References

[1] Brewer NT , Chapman GB, Rothman AJ, Leask J, Kempe A. Increasing vaccination: Putting psychological science into action. Psychol Sci Public Interest. 2017;18:149-207. 10.1177/1529100618760521 29611455

[2] Larson HJ , Gakidou E, Murray CJL. The vaccine-hesitant moment. N Engl J Med. 2022;387:58-65. 10.1056/NEJMra2106441 35767527 PMC9258752

[3] World Health Organization. Ten Health Issues WHO Will Tackle This Year. 2019. https://www.who.int/news-room/spotlight/ten-threats-to-global-health-in-2019

[4] Baldwin AS , Tiro JA, Zimet GD. Broad perspectives in understanding vaccine hesitancy and vaccine confidence: an introduction to the special issue. J Behav Med. 2023;46:1-8. 10.1007/s10865-023-00397-8 36802315 PMC9942647

[5] Increase the Proportion of People Who Get the Flu Vaccine Every Year–IID‑09—Healthy People 2030 | Health.gov. https://health.gov/healthypeople

[6] CDC. Flu Vaccination Coverage Update for the 2023-2024 Season. Centers for Disease Control and Prevention. February 23, 2024. https://www.cdc.gov/flu/spotlights/2023-2024/vaccination-coverage-update.htm

[7] Brewer NT , DeFrank JT, Gilkey MB. Anticipated regret and health behavior: a meta-analysis. Health Psychol. 2016;35:1264-1275. 10.1037/hea0000294 27607136 PMC5408743

[8] Sandberg T , Conner M. Anticipated regret as an additional predictor in the theory of planned behaviour: a meta-analysis. Br J Soc Psychol. 2008;47:589-606. 10.1348/014466607X258704 18039428

[9] Michie S , Richardson M, Johnston M, et al The behavior change technique taxonomy (v1) of 93 hierarchically clustered techniques: building an international consensus for the reporting of behavior change interventions. Ann Behav Med. 2013;46:81-95. 10.1007/s12160-013-9486-6 23512568

[10] Brewer NT , Gottlieb SL, Reiter PL, et al Longitudinal predictors of human papillomavirus vaccine initiation among adolescent girls in a high-risk geographic area. Sex Transm Dis. 2011;38:197-204. 10.1097/OLQ.0b013e3181f12dbf 20838362 PMC3025264

[11] Chapman GB , Coups EJ. Emotions and preventive health behavior: worry, regret, and influenza vaccination. Health Psychol. 2006;25:82-90. 10.1037/0278-6133.25.1.82 16448301

[12] Sheeran P , Klein WMP, Rothman AJ. Health behavior change: moving from observation to intervention. Annu Rev Psychol. 2017;68:573-600. 10.1146/annurev-psych-010416-044007 27618942

[13] Riddle M ; Science of Behavior Change Working Group. News from the NIH: using an experimental medicine approach to facilitate translational research. Transl Behav Med. 2015;5:486-488. 10.1007/s13142-015-0333-0 26622921 PMC4656226

[14] Rothman AJ , Baldwin AS. A person x intervention strategy approach to understanding health behavior. In: K Deaux, M Snyder, eds. Handbook of Personality and Social Psychology. 2nd ed. Oxford University Press; 2019:831-856.

[15] Rothman AJ , Sheeran P. The operating conditions framework: integrating mechanisms and moderators in health behavior interventions. Health Psychol. 2021;40:845-857. 10.1037/hea0001026 32914997

[16] Cox D , Sturm L, Cox AD. Effectiveness of asking anticipated regret in increasing HPV vaccination intention in mothers. Health Psychol. 2014;33:1074-1083. 10.1037/hea0000071 24611739

[17] Sandberg T , Conner M. A mere measurement effect for anticipated regret: impacts on cervical screening attendance. Br J Soc Psychol. 2009;48:221-236. 10.1348/014466608X347001 18793492

[18] Abraham C , Sheeran P. Deciding to exercise: the role of anticipated regret. Br J Health Psychol. 2004;9:269-278. 10.1348/135910704773891096 15125809

[19] O'Carroll RE , Chambers JA, Brownlee L, Libby G, Steele RJC. Anticipated regret to increase uptake of colorectal cancer screening (ARTICS): a randomised controlled trial. Soc Sci Med. 2015;142:118-127. 10.1016/j.socscimed.2015.07.026 26301484 PMC4576211

[20] O'Carroll RE , Dryden J, Hamilton-Barclay T, Ferguson E. Anticipated regret and organ donor registration—a pilot study. Health Psychol. 2011;30:661-664. 10.1037/a0024182 21644807

[21] Ryan RM , Deci EL. Self-determination theory and the facilitation of intrinsic motivation, social development, and well-being. Am Psychol. 2000;55:68-78. 10.1037/0003-066X.55.1.68 11392867

[22] Ryan RM , Patrick H, Deci EL, Williams GC. Facilitating health behaviour change and its maintenance: interventions based on self-determination theory. Eur Health Psychol. 2008;10:2-5.

[23] Gorin AA , Powers TA, Koestner R, Wing RR, Raynor HA. Autonomy support, self-regulation, and weight loss. Health Psychol. 2014;33:332-339. 10.1037/a0032586 23730718 PMC5330668

[24] Ntoumanis N , Ng JYY, Prestwich A, et al A meta-analysis of self-determination theory-informed intervention studies in the health domain: effects on motivation, health behavior, physical, and psychological health. Health Psychol Rev. 2021;15:214-244. 10.1080/17437199.2020.1718529 31983293

[25] Connolly T , Zeelenberg M. Regret in decision making. Curr Dir Psychol Sci. 2002;11:212-216. 10.1111/1467-8721.00203

[26] Zeelenberg M , Pieters R. A theory of regret regulation 1.0. J Consum Psychol. 2007;17:3-18. 10.1207/s15327663jcp1701_3 PMC2435165 18568095

[27] Wrosch C , Heckhausen J. Perceived control of life regrets: good for young and bad for old adults. Psychol Aging. 2002;17:340-350. 10.1037/0882-7974.17.2.340 12061416

[28] Wrosch C , Bauer I, Scheier MF. Regret and quality of life across the adult life span: the influence of disengagement and available future goals. Psychol Aging. 2005;20:657-670. 10.1037/0882-7974.20.4.657 16420140

[29] Levesque CS , Williams GC, Elliot D, Pickering MA, Bodenhamer B, Finley PJ. Validating the theoretical structure of the Treatment Self-Regulation Questionnaire (TSRQ) across three different health behaviors. Health Educ Res. 2007;22:691-702. 10.1093/her/cyl148 17138613

[30] Teixeira PJ , Marques MM, Silva MN, et al A classification of motivation and behavior change techniques used in self-determination theory-based interventions in health contexts. Motiv Sci. 2020;6:438-455. 10.1037/mot0000172

[31] Fall E , Izaute M, Chakroun-Baggioni N. How can the health belief model and self-determination theory predict both influenza vaccination and vaccination intention? A longitudinal study among university students. Psychol Health. 2018;33:746-764. 10.1080/08870446.2017.1401623 29132225

[32] Baldwin AS , Zhu H, Rochefort C, et al Mechanisms of self-persuasion intervention for HPV vaccination: testing memory and autonomous motivation. Health Psychol. 2021;40:887-896. 10.1037/hea0001075 34138615 PMC8678358

[33] Schmitz M , Luminet O, Klein O, et al Predicting vaccine uptake during COVID-19 crisis: a motivational approach. Vaccine. 2022;40:288-297. 10.1016/j.vaccine.2021.11.068 34961635 PMC8626229

[34] Baldwin AS , Rothman AJ, Vander Weg MW, Christensen AJ. Examining causal components and a mediating process underlying self-generated health arguments for exercise and smoking cessation. Health Psychol. 2013;32:1209-1217. 10.1037/a0029937 23025303

[35] Weinstein ND , Kwitel A, McCaul KD, Magnan RE, Gerrard M, Gibbons FX. Risk perceptions: assessment and relationship to influenza vaccination. Health Psychol. 2007;26:146-151. 10.1037/0278-6133.26.2.146 17385965

[36] Denman DC , Baldwin AS, Marks EG, Lee SC, Tiro JA. Modification and validation of the Treatment Self Regulation Questionnaire to assess parental motivation for HPV vaccination of adolescents. Vaccine. 2016;34:4985-4990. 10.1016/j.vaccine.2016.08.037 27595447 PMC5028302

[37] Irving SA , Donahue JG, Shay DK, Ellis-Coyle TL, Belongia EA. Evaluation of self-reported and registry-based influenza vaccination status in a Wisconsin cohort. Vaccine. 2009;27:6546-6549. 10.1016/j.vaccine.2009.08.050 19729083

[38] Mangtani P , Shah A, Roberts JA. Validation of influenza and pneumococcal vaccine status in adults based on self-report. Epidemiol Infect. 2007;135:139-143. 10.1017/S0950268806006479 16740194 PMC2870540

[39] Valeri L , VanderWeele TJ. Mediation analysis allowing for exposure–mediator interactions and causal interpretation: theoretical assumptions and implementation with SAS and SPSS macros. Psychol Methods. 2013;18:137-150. 10.1037/a0031034 23379553 PMC3659198

[40] Valente MJ , Rijnhart JJM, Smyth HL, Muniz FB, MacKinnon DP. Causal mediation programs in R, M plus, SAS, SPSS, and stata. Struct Equ Model Multidiscip J. 2020;27:975-984. 10.1080/10705511.2020.1777133 PMC7853644 33536726

[41] MacKinnon DP , Valente MJ, Gonzalez O. The correspondence between causal and traditional mediation analysis: the link is the mediator by treatment interaction. Prev Sci. 2020;21:147-157. 10.1007/s11121-019-01076-4 31833021 PMC6992469

[42] Xu S , Coffman DL, Luta G, Niaura RS. Tutorial on causal mediation analysis with binary variables: an application to health psychology research. Health Psychol. 2023;42:778-787. 10.1037/hea0001299 37410423 PMC10615709

[43] Imai K , Keele L, Yamamoto T. Identification, inference and sensitivity analysis for causal mediation effects. Statist. Sci. 2010;25:51-71. 10.1214/10-STS321

[44] Liao Q , Wong WS, Fielding R. How do anticipated worry and regret predict seasonal influenza vaccination uptake among Chinese adults? Vaccine. 2013;31:4084-4090. 10.1016/j.vaccine.2013.07.009 23867015

[45] Sheeran P , Wright CE, Avishai A, et al Self-determination theory interventions for health behavior change: meta-analysis and meta-analytic structural equation modeling of randomized controlled trials. J Consult Clin Psychol. 2020;88:726-737. 10.1037/ccp0000501 32437175

[46] Sheeran P , Wright CE, Avishai A, Villegas ME, Rothman AJ, Klein WMP. Does increasing autonomous motivation or perceived competence lead to health behavior change? A meta-analysis. Health Psychol. 2021;40:706-716. 10.1037/hea0001111 34881939

[47] O'Carroll RE , Shepherd L, Hayes PC, Ferguson E. Anticipated regret and organ donor registration: a randomized controlled trial. Health Psychol. 2016;35:1169-1177. 10.1037/hea0000363 27280372

[48] Abraham C , Sheeran P. Acting on intentions: the role of anticipated regret. Br J Soc Psychol. 2003;42:495-511. 10.1348/014466603322595248 14715114

[49] Ferrer RA , Cohen GL. Reconceptualizing self-affirmation with the trigger and channel framework: lessons from the health domain. Personal Soc Psychol Rev. 2019;23:285-304. 10.1177/1088868318797036 30295141

[50] Sheeran P , Suls J, Bryan A, et al Activation versus change as a principle underlying intervention strategies to promote health behaviors. Ann Behav Med. 2023;57:205-215. 10.1093/abm/kaac045 36082928 PMC10305802

