# Supplementary material for: Testing autonomous motivation as a mechanism of anticipated regret intervention techniques for seasonal flu vaccination
Source: Ann Behav Med. 2025 Sep 2;59(1):kaaf053. doi: 10.1093/abm/kaaf053 (PMC12397997; doi:10.1093/abm/kaaf053)
Supplement: kaaf053_Supplementary_Data [file kaaf053_supplementary_data.zip › Supplemental Materials.docx]

Supplemental Figure 1. Timing of study assessments.


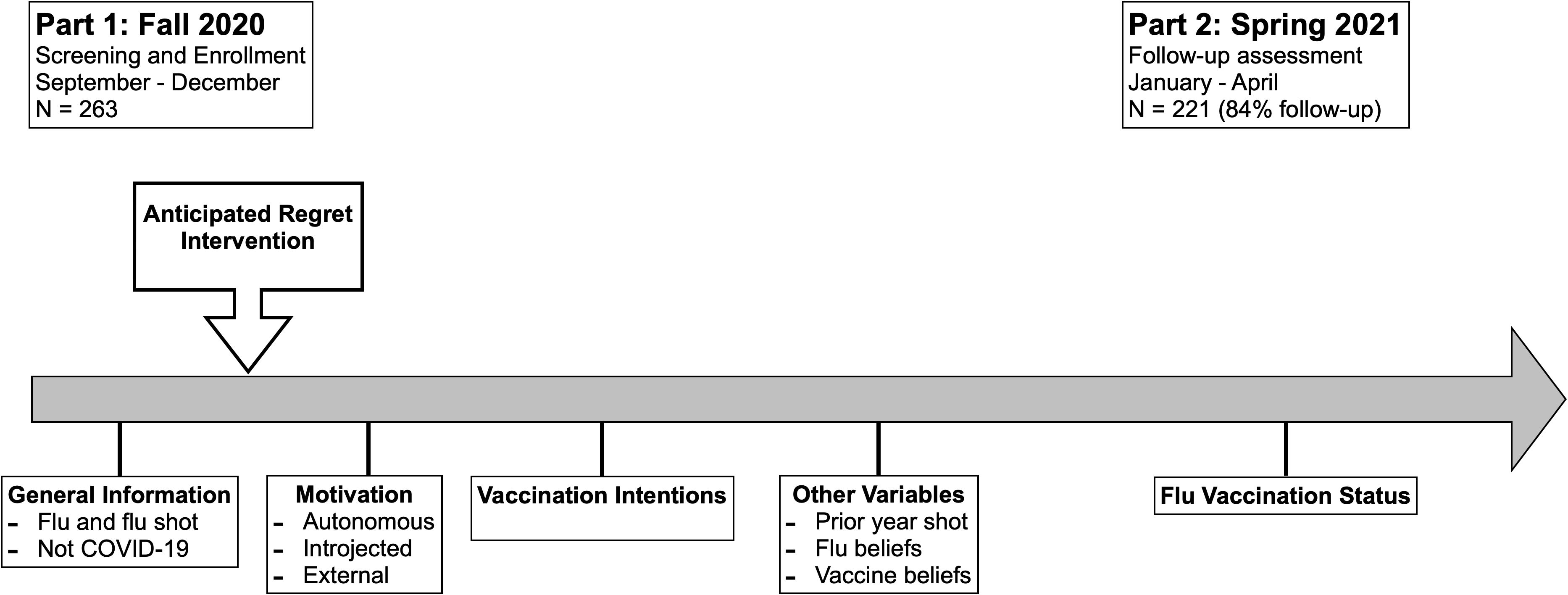


Supplemental Table 1. Results of models excluding participants with very fast response times (< 1 second/item) on motivation items (n=4) and intention items (n=1).

| Analyses involving motivation |  |
| --- | --- |
| Anticipated regret (AR) on autonomous motivation |  |
| Self-generated AR vs. control | ***β* = .184, *t* = 2.84, *p* = .005** |
| Mere measurement AR vs. control | *β* = .049, *t* = 0.76, *p* = .45 |
| Self-generated AR vs. mere measurement | ***β* = .135, *t* = 2.07, *p* = .04** |
| AR on introjected motivation | *βs <* .09, *p*s > .20 |
| AR on external motivation | *βs <* .12, *p*s > .10 |
| Indirect effect on flu shot intentions |  |
| ACME: Self-generated AR vs. control | **estimate: 0.331, 95% CI: 0.119, 0.56, *p* = .004** |
| ACME: Mere measurement of AR vs. control | estimate: 0.089, 95% CI: -0.154, 0.34, *p* = .47 |
| Indirect effect on vaccination |  |
| ACME: Self-generated AR vs. control | **estimate: 0.034, 95% CI: 0.007, 0.08, *p* = .006** |
| ACME: Mere measurement of AR vs. control | estimate: 0.013, 95% CI: -0.010, 0.05, *p* = .29 |
| Analyses involving flu shot intentions |  |
| AR on intentions |  |
| Self-generated AR vs. control | *β* = .067, *t* = 1.07, *p* = .29 |
| Mere measurement AR vs. control | *β* = -.056, *t* = -.888, *p* = .38 |
| Self-generated AR vs. mere measurement | ***β* = .124, *t* = 1.95, *p* = .05** |

Note. Bold values indicate statistically significant effects. The pattern of significant and non-significant effect is the same as the findings reported in the text of the paper. ACME = average causal mediated effect; AR = anticipated regret; CI = confidence interval.

Supplemental Table 2. Correlations and descriptive statistics for anticipated regret items in the mere measurement condition (n = 88)

| Variables | 1 | 2 | 3 | 4 | 5 | 6 |
| --- | --- | --- | --- | --- | --- | --- |
| 1. I would regret it if I did not get a flu shot and later got sick with the flu. | - |  |  |  |  |  |
| 2. If I did not get a flu shot and later got sick with the flu, I would wish I had. | .819 | - |  |  |  |  |
| 3. …have to spend several days in bed with unpleasant symptoms? | .622 | .620 | - |  |  |  |
| 4. …end up spreading the flu to others? | .481 | .438 | .673 | - |  |  |
| 5. …miss several of your classes? | .443 | .383 | .643 | .533 | - |  |
| 6. …have to skip social activities? | .394 | .387 | .535 | .438 | .502 | - |
| Variables |  |  |  |  |  |  |
| M | 3.51 | 3.48 | 3.31 | 3.32 | 3.31 | 3.19 |
| SD | 1.32 | 1.36 | 0.94 | 0.92 | 0.93 | 0.93 |

Note. Items 3-6 all included the following sentence stem: “Imagine you get sick later in the year with the flu, but a flu shot would have prevented it. How much would you regret that you did not get a flu shot if you…” followed by each specific negative consequence. The response scales for items 1 and 2 was 1 (*strongly disagree*) to 5 (*strongly agree*) and for items 3-6 was 1 (*Not a lot*) to 4 (*A lot*).

Supplemental Table 3. Correlations between measures of anticipated regret and motivation types in the mere measurement condition.

| Variables | 1 | 2 | 3 | 4 | 5 |
| --- | --- | --- | --- | --- | --- |
| 1. Anticipated regret (two items: general consequences) | - |  |  |  |  |
| 2. Anticipated regret (four items: specific consequences) | .605 | - |  |  |  |
| 3. Autonomous motivation | .689 | .667 | - |  |  |
| 4. Introjected motivation | .500 | .430 | .502 | - |  |
| 5. External motivation | .180 | .196 | .182 | .496 | - |

Note. The bivariate correlations indicate that anticipated regret is associated with both autonomous and introjected motivations, but more strongly with autonomous motivation. This pattern of correlations is consistent with the intervention effects reported in the paper.

Supplemental Table 4. Logistic regressions testing association between autonomous motivation and vaccination.

|  | B | S.E. | *p* | Odds Ratio (OR) | 95 % CI for OR |
| --- | --- | --- | --- | --- | --- |
| Model 1 |  |  |  |  |  |
| Prior year vaccination | 1.257 | .335 | <.001 | **3.52** | **1.82, 6.78** |
| Autonomous motivation | .578 | .205 | .005 | **1.78** | **1.19, 2.66** |
| Model 2 |  |  |  |  |  |
| Prior year vaccination | 1.009 | .351 | .004 | **2.74** | **1.38, 5.46** |
| Autonomous motivation | -.162 | .281 | .564 | 0.85 | 0.49, 1.48 |
| Flu shot intentions | .909 | .238 | <.001 | **2.48** | **1.56, 3.96** |

Note. Model 1 tests the association of autonomous motivation on vaccination controlling for prior year vaccination status. Model 2 adds flu shot intentions, the initial outcome assessed, to the model. The results of Model 2 indicate that autonomous motivation does not predict vaccination when intentions are included in the model. Bold values indicate statistically significant associations.
